# Predicting the protein localization sites using artificial neural networks

**DOI:** 10.1186/1758-2946-5-S1-P46

**Published:** 2013-03-22

**Authors:** V Arulmozhi, Rajesh Reghunadhan

**Affiliations:** 1 Dept. of Computer Appl., Tirupur Kumaran College for Women, India; 2 Dept. of Computer Appl., Bharathiar University, Coimbatore - 641046, India

## 

Chemoinformatics, the brain child of Frank Brown [1], has now evolved into a new branch of science, which has high correlations with computer science, bioinformatics, and chemistry. The major functionalities of Chemoinformatics include, but not limited to, chemical structure/property prediction, molecular similarity/diversity analysis, virtual screening, qualitative/quantitative structural/activity/property relationship, design of combinatorial libraries, statistical models, descriptors, drug discovery, representation of chemical compounds/reactions, classification/search/storage methods, management of compound databases, high-throughput docking, data analysis methods, etc. This paper deals with the prediction of localization sites of protein using neural network.

Neural Network [2] provides learning capability and it is one of the important components of softcomputing. A neural network will consist of one input layer, one or more number of hidden layers and an output layer. Number of neurons in the input layer will be equal to the number of features passed to the neural network. Number of neurons in the output layer will be equal to the number of classes for classification purpose. Hidden neurons are usually fixed by experts depending on the problem. There are various types of neural network available like feedforward neural networks, feedback networks, reccurrent networks, self organizing maps, anfis, etc.

In this paper E.coli protein dataset [3] is used for prediction. The data set with 336 instances is having 7 attributes with 8 classes (localization sites). The dataset can be obtained from UCI machine repository. Neural network with 500 hidden neurons and scaled conjugate gradient algorithm are used in this work. The classification result shown in the table 1 for our method, is the average of 4 cross validation and the results are promising.

**Table 1:** Classification rates.

Probabilistic classification [3]	81.1%
Decision Tree [4]	80.36%
Naïve Bayes [4]	80.95%
Our neural network method	83.33%
